# Electrochemical Fingerprints of Illicit Drugs on Graphene and Multi-Walled Carbon Nanotubes

**DOI:** 10.3389/fchem.2021.641147

**Published:** 2021-03-16

**Authors:** Ana-Maria Dragan, Florina Maria Truta, Mihaela Tertis, Anca Florea, Jonas Schram, Andreea Cernat, Bogdan Feier, Karolien De Wael, Cecilia Cristea, Radu Oprean

**Affiliations:** ^1^ Department of Analytical Chemistry and Instrumental Analysis, “Iuliu Haţieganu” University of Medicine and Pharmacy, Cluj-Napoca, Romania; ^2^ Axes Research Group, University of Antwerp, Antwerp, Belgium; ^3^ NANOlab Center of Excellence, University of Antwerp, Antwerp, Belgium

**Keywords:** cocaine, MDMA, MMC, PVP, graphene, MWCNT, electrochemical sensors

## Abstract

Illicit drugs use and abuse remains an increasing challenge for worldwide authorities and, therefore, it is important to have accurate methods to detect them in seized samples, biological fluids and wastewaters. They are recently classified as the latest group of emerging pollutants as their consumption increased tremendously in recent years. Nanomaterials have gained much attention over the last decade in the development of sensors for a myriad of applications. The applicability of these nanomaterials, functionalized or not, significantly increases and it is therefore highly suitable for use in the detection of illicit drugs. We have assessed the suitability of various nanoplatforms, such as graphene (GPH), multi-walled carbon nanotubes (MWCNTs), gold nanoparticles (AuNPs) and platinum nanoparticles (PtNPs) for the electrochemical detection of illicit drugs. GPH and MWCNTs were chosen as the most suitable platforms and cocaine, 3,4-methylendioxymethamfetamine (MDMA), 3-methylmethcathinone (MMC) and α-pyrrolidinovalerophenone (PVP) were tested. Due to the hydrophobicity of the nanomaterials-based platforms which led to low signals, two strategies were followed namely, pretreatment of the electrodes in sulfuric acid by cyclic voltammetry and addition of Tween 20 to the detection buffer. Both strategies led to an increase in the oxidation signal of illicit drugs. Binary mixtures of illicit drugs with common adulterants found in street samples were also investigated. The proposed strategies allowed the sensitive detection of illicit drugs in the presence of most adulterants. The suitability of the proposed sensors for the detection of illicit drugs in spiked wastewaters was finally assessed.

## Introduction

In the last years, illicit drug consumption has increased tremendously and it seriously affects the public health worldwide (United Nations Publications, 2020). This fact is linked to the European drug market which continues to evolve and to produce new psychoactive substances that are very difficult to detect by the authorities. Recent changes in the drug market are associated with technology development and globalization. These changes include innovation in drug production, new trafficking methods, and the growth of online markets (EMCDDA, 2020). The abuse of these substances has severe consequences for our society, increased health costs, economic impact, increasing criminality, and environment pollution (Florea et al., 2018b).

After cannabis, cocaine is the second most used illicit drug in Europe and one of the most consumed illicit drugs (Florea et al., 2018b; De Jong et al., 2018; EMCDDA, 2020). This alkaloid drug is highly addictive because it stimulates the central nervous system and causes euphoria and dependence, and at the same time is very harmful for people’s health causing an increase in blood pressure, heart rate and respiration rate. When the regular consumption is stopped the lethargy could appear, followed by depression and extreme tiredness (Samyn et al., 2009; De Jong et al., 2016). Another illicit substance that is studied in the present work is MDMA, the psychoactive compound in Ecstasy. MDMA is a synthetic drug that is chemically related to amphetamine and is used as a recreational drug, because of its very powerful stimulation effects on the nervous system (EMCDDA, 2020). The problem is that this drug is very often misused which can lead to sever health problems because of its neurotoxic effects (Narang et al., 2018). MMC is a structural isomer of mephedrone, and it is a new designer drug from the substituted cathinones family, that became popular as cheap substitutes for traditional drugs and because their structures could circumvent legislation. This drug is very often used in combination with other substances in order to enhance the euphoric experiences (Jamey et al., 2016). This drug has similar psychostimulant properties to amphetamines, including euphoria, alertness, physical energy, feelings of empathy and awareness (Dias da Silva et al., 2019). PVP is a synthetic cathinone belonging to the group of “second generation” pyrrolidinophenones (Grapp et al., 2016). PVP has powerful cocaine-like stimulant effects, a high brain penetration, and a high liability for abuse. Its adverse effects could include tachycardia, agitation, hypertension, hallucinations, delirium, and even suicidal ideations (Nóbrega and Dinis-Oliveira, 2018). The rise of these so called designer drugs and the fact that the synthetic drug market is full of the structural analogues of these two cathinones represent justifies for the selection of MMC and PVP to be analyzed in this study.

For the detection of these drugs numerous techniques were applied. Among them are gas chromatography coupled with mass spectrometry (GC–MS), high-performance liquid chromatography (HPLC), liquid chromatography-mass spectrometry (LC-MS), ion mobility spectrometry (IMS), capillary electrophoresis (CE), and immunoassays. Unfortunately, these techniques present some disadvantages like high cost, complicated operations, and lengthy analysis time (Shen et al., 2015). Electrochemical methods, on the other hand, offer a fast, portable, low-cost, and accurate alternative for the analysis of forensic drugs and their metabolites (Florea et al., 2018b).

Nowadays, of great concern regarding the illicit seizures and samples is their composition since other substances, besides the drug itself, are added to the products sold on the illicit market. These substances are called cutting agents and they can be classified as follows: (i) bulk agents, which are added as fillers, (ii) adulterants, which are meant to suppress the adverse effects of the drug, to mimic and enhance the desired ones or to facilitate the administration, and (iii) other illicit drugs (Cole et al., 2011). The most common bulk agents used in the drug samples are starch, sugars like lactose or mannitol, but also acetaminophen and caffeine which can be considered both adulterants and bulk agents. Other adulterants encountered in drug samples are lidocaine, procaine, creatine, phenacetine, levamisole and diltiazem (Kudlacek et al., 2017; Fiorentin et al., 2019). While bulk agents like starch or lactose do not have great effects on the health of the consumers, the adulterants can increase the severity of the effects induced by the illicit drugs and the risk of mortality, especially because the proportion of drugs and adulterants in the samples presents wide variations, from pure drug samples to the extent of no drug at all (Kudlacek et al., 2017). Besides their toxicological implications, these substances can interfere with the detection and analysis of the illicit drugs, therefore their investigation is very important from an analytical point of view. The main issue is the fact that the oxidation signal of the drugs could be overlapped by other adulterants generating a false negative or a false positive response if the adulterant generates a peak in the same potential zone as the drug.

Since there were several studies conducted for the electrochemical detection of cocaine samples adulterated with levamisole (Florea et al., 2018a; De Jong et al., 2018), our study focused on some of the other common cutting agents encountered in drug samples, namely caffeine, acetaminophen, lactose and benzocaine.

In order to improve sensitivity and selectivity, the screen printed electrodes (SPEs) can be modified with carbon-based nanomaterials, such as GPH and MWCNTs or metal NPs, such as AuNPs, PtNPs and silver (Florea et al., 2018a; Ahmed et al., 2020). These nanomaterials provide higher surface area, increased conductivity and faster electron transfer (Eissa et al., 2019; De Rycke et al., 2020).

Herein, we report an extensive study that evaluates the influence of different platforms with carbon/metallic NPs on the detection of illicit drugs, namely cocaine, MDMA, MMC, and PVP, as single analytes and binary mixtures with adulterants mentioned in the literature as the most commonly used compounds for this purpose. Two electrolytic media with different pH values were used, two different pretreatment strategies were also tested and real samples were successfully analyzed in order to have enough insights for the detection of illicit drugs in real scenarios.

## Materials and Methods

### Materials and Instrumentation

All chemicals used in this study were of analytical grade and were used as received from the manufacturer without further purification. All solutions used were prepared with ultrapure water (18.2 MΩ, Millipore Simplicity). Cocaine, methamphetamine, MDMA, MMC, PVP where purchased from Cayman Chemicals, while, acetaminophen, benzocaine, K_2_HPO_4_, KH_2_PO_4_, KCl, H_2_PtCl_6_, HCl, HAuCl_4_, H_2_SO_4_, [Fe(CN)_6_]^4-/3-^, NaOH, Tween 20 were purchased from Merck. Phosphate buffer saline (PBS) solution of 0.1 M with 0.1 M KCl was used as the supporting electrolyte and it was prepared with K_2_HPO_4_ and KH_2_PO_4_, adjusted to the mentioned values of pH with either NaOH or HCl.

The electrochemical experiments were performed using an AUTOLAB PGSTAT 302N (EcoChemie, Netherlands) equipped with the associated NOVA 1.10 software. All the SPEs with a silver pseudo reference, a carbon counter electrode and with different working electrodes: graphite-based, graphite-based modified with GPH and MWCNTs, were provided by Metrohm (Spain). The data analysis and the creation of figures were performed using the Origin 8.5 software (OriginLab, United States). For a better visualization, all the SWV voltammograms presented here were baseline-corrected using the moving average filter included in the NOVA 1.10 software (window size 1), without affecting the results.

### Elaboration of the Nanomaterials-Based Platforms

Bare graphite SPEs were modified in the lab with the following nanomaterials: (i) PtNPs); (ii) AuNPs using the following methods:

- PtNPs modification: PtNPs were electrochemically deposited directly on the surface of graphite SPEs from a solution of 10 mM H_2_PtCl_6_ in 0.1 M HCl *via* cyclic voltammetry (CV) from -0.3 V to 1.4 V for 20 cycles with a scan rate of 100 mV/s;
- AuNPs modification: AuNPs were electrochemically deposited directly on the surface of graphite SPEs from a solution of 5 mM HAuCl_4_ in 0.1 M H_2_SO_4_ by CV from -0.2 V to 1.4 V, 20 cycles, with a scan rate of 50 mV/s.

### Platforms Characterization

An electrochemical characterization of SPE platforms was performed by CV and electrochemical impedance spectroscopy (EIS). CV was performed in 5 mM [Fe(CN)_6_]^4-/3-^ solution in 0.1 M KCl in the potential window from -0.5 V to 0.8 V with a scan rate of 100 mV/s. EIS tests were performed in 5 mM [Fe(CN)_6_]^4-/3-^ solution in 0.1 M KCl from 100,000 to 0.1 Hz, with 61 frequencies applied and an amplitude of 0.01 V.

### Electrochemical Fingerprint of Illicit Drugs/Adulterants by SWV

The electrochemical fingerprint of the illicit drugs and adulterants/cutting agents was studied using square wave voltammetry (SWV) performed in the potential window from 0 to 1.3 V with a step potential of 5 mV, an amplitude of 25 mV and a frequency of 10 Hz in the following solutions: (i) 0.5 mM of illicit drug; (ii) 0.5 mM of adulterant; (iii) binary mixtures of illicit drug : adulterant 0.5 mM : 0.5 mM. All these solutions were prepared in PBS.

The tests were performed on the following platforms: (i) graphite-based SPEs, (ii) commercially available graphite SPEs modified with GPH (SPE-GPH), (iii) commercially available graphite SPE modified with MWCNTs (SPE-MWCNTs); (iv) graphite SPEs modified with PtNPs (SPE-PtNPs) and (v) with AuNPs (SPE-AuNPs) as described in *Elaboration of the Nanomaterials-Based Platforms* section.

### (Pre)Treatment Strategies

Two separate strategies were employed as an attempt to increase the electrochemical signal of the drugs:

- A pretreatment with 0.5 M H_2_SO_4_ solution was applied on the electrodes by CV for 5 cycles in the potential window from -0.5 V to 1.5 V with a step potential of 2.44 mV and a scan rate of 100 mV/s before the SWV was performed.
- Modification of the electrolyte composition by adding 1% Tween 20, a polysorbate surfactant, in the analyzed solutions.

### Real Samples

The optimized method was applied to real samples consisting of tap and waste water diluted in 1:1 ratio with the supporting electrolyte (PBS of chosen pH) and spiked with the studied illicit drugs in order to reach a concentration of 0.5 mM. After the dilution, the pH of the sample was verified and adjusted to the desired value.

## Results and Discussion

In order to maximize the outcome of our study, we followed a systematic approach and assessed the influence of the support and pH on the electrochemical behavior of the set of targets. After that, the characterization via CV and EIS was performed on the chosen platforms prior to the pretreatment steps (H_2_SO^4^ and Tween 20 conditioning) meant to improve the analytical signal. The next step consists in the evaluation of the adulterants and their binary mixtures with the targets in the optimized conditions. Finally, real samples consisting in waste water and tap water were analyzed with the optimized method.

### Influence of the Platform Composition and the pH of the Electrolyte

**FIGURE 1:**
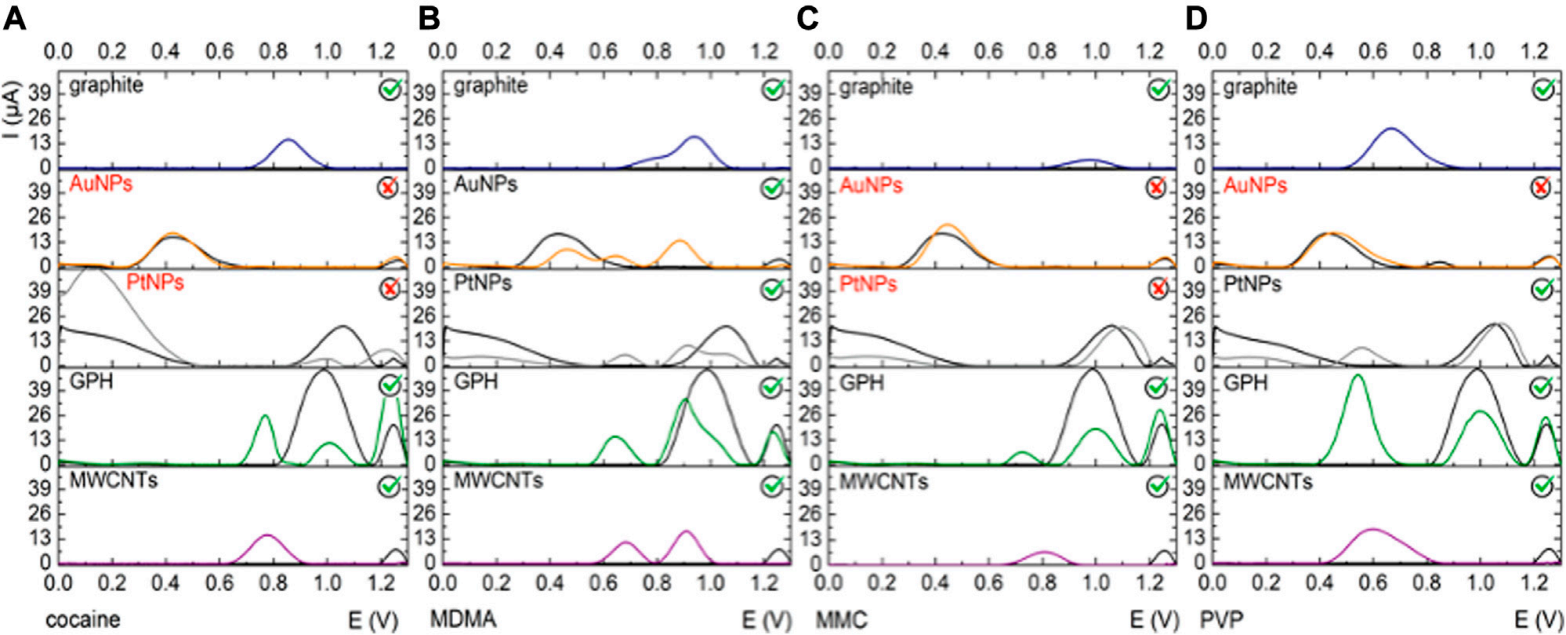
Electrochemical fingerprints of the drugs on different platforms: (i) graphite-SPE (blue); (ii) SPE-AuNPs (orange); (iii) SPE-PtNPs (gray); (iv) SPE-GPH (green); (v) SPE-MWCNTs (purple) in 0.5 mM solution of each illicit drug in PBS pH 12: **(A)** cocaine, **(B)** MDMA, **(C)** MMC and **(D)** PVP and the blanks with black.

The first optimization step consisted in the assessment of the influence of the studied platforms on both illicit drug molecules as it can be seen from the data presented in Table 1. The SWVs at pH 12 are presented below in Figure 1 [the data were baseline corrected using the moving average filter from Nova 1.10 software (window size 1)].

**TABLE 1:** The influence of the type of platform and pH on the electrochemical oxidation of illicit drugs. The data in bold represent the highest values of the current intensity for one analyte.

Platform	pH	Cocaine		MDMA		MMC		PVP	
		E (V)	I (µA)	E (V)	I (µA)	E (V)	I (µA)	E (V)	I (µA)
Graphite	7	0.99	4.099 ± 0.09	1.05	20.64 ± 1.23	1.04	1.28 ± 0.02	0.77	12.22 ± 0.83
	**12**	**0.85**	**14.93** ± 1.25	**0.94**	**16.56** ± 0.84	**0.97**	**4.15** ± 0.04	**0.66**	**20.78** ± 0.89
AuNPs	7	—	—	0.99	280 ± 14.14	—	—	—	—
	12	—	—	0.88	13.56 ± 0.41	—	—	—	—
PtNPs	7	0.94	0.37 ± 0.02	0.96	29.93 ± 1.21	—	—	0.73	3.56 ± 0.15
	12	—	—	0.68	5.73 ± 0.09	—	—	0.55	9.23 ± 0.43
**GPH**	7	0.81	14.59 ± 0.49	0.97	61.53 ± 0.72	0.85	7 ± 0.62	0.75	3.37 ± 0.19
	**12**	**0.77**	**26.07** ± 0.75	**0.63**	**14.81** ± 1.26	**0.72**	**6.57** ± 0.36	**0.54**	**47.33** ± 0.94
**MWCNTs**	7	0.931	11.07 ± 0.08	0.97	31.16 ± 0.48	0.900	9.26 ± 0.97	0.72	17.17 ± 0.55
	**12**	**0.77**	**15.04** ± 0.91	**0.68** **0.91**	**10.82** ± 0.49 **16.69** ± 0.91	**0.81**	**6.76** ± 0.46	**0.59**	**17.98** ± 1.40

As it can be seen from Table 1, the higher current intensity for the studied molecules was obtained on the unmodified graphite electrode, GPH and MWCNTs electrodes. Despite the fact that the current intensity was higher in some cases on the AuNPs-based platform at pH 7, at pH 12 a signal was observed only for MDMA, and not for the other compounds.

On the graphite electrodes modified with AuNPs at pH 12 only MDMA had a characteristic analytical signal, while on PtNPs only MDMA and PVP were detected at the same pH value. Also the intensity of the electrooxidation signals was lower than the ones obtained on the other platforms suggesting that the electron transfer rates were low. In this case, the optimization continued only on graphite, GPH and MWCNTs electrodes. When assessing the analytical signal of the compounds depending on the pH in the majority of cases the potential had a cathodic shift and the current intensity was higher at pH 12. These changes are expectedly observed on every platform because the oxidation reactions of the majority of the studied compounds involve protons and a more alkaline pH facilitates these reactions. The exception is MDMA since its main peak is caused by the oxidation of the aromatic nucleus, which doesn’t involve protons. Each compound was individually studied, as follows: (1) for cocaine, MDMA and MMC the best results in terms of current intensity were obtained on GPH and MWCNTs at pH 12 suggesting an improvement in the electronic transfer rate due to the presence of nanomaterials. Also, a cathodic shift for the electrochemical oxidation potential was clearly visible; (2) when studying the electrochemical behavior of PVP, similar results were obtained as described above, only the signal on MWCNTs was lower than the one obtained on the unmodified graphite electrodes.

In light of the previewed applications that consist in the simultaneous detection based on the current intensity and potential value of the majority of compounds, the optimal pH value was considered to be pH 12 on GPH and MWCNTs electrodes (Figure 1) (De Jong et al., 2018). Thus, all the following experiments were performed on graphite-based SPEs functionalized with GPH and MWCNTs.

### Characterization of the Platforms via Electrochemical Methods

**FIGURE 2:**
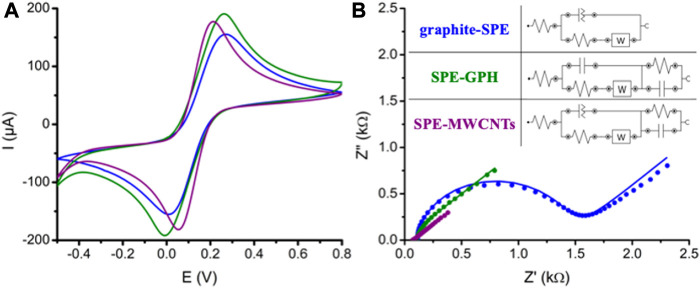
Characterization of the graphite-SPE (blue), SPE-GPH (green), SPE-MWCNTs (purple) via **(A)** CV and **(B)** EIS in 5 Fe(CN)_6_]^4-/3-^ solution in 0.1 M KCl (CV: from -0.5 V to 0.8 V with a scan rate of 100 mV/s; EIS: a frequency of 100,000 to 0.1 Hz with 61 points and an amplitude of 0.01 V); Inset equivalent circuits of the studied platforms.

The platforms were characterized via electrochemical methods namely CV and EIS to evaluate the electron transfer rate and the impact of the modifiers on its variation (Figure 2).

The CV clearly showed an increase in the current intensity corresponding to the redox couple of [Fe(CN)_6_]^3−/4−^ on the GPH electrode in comparison with the graphite-based one. When the MWCNT-based platform data was assessed a slight decrease in the current intensity was observed in comparison with the GPH platform and also a cathodic/anodic shift corresponding to the oxidation/reduction peaks of the redox couple (Figure 2A). This fact suggest an improvement of the electron transfer rate on the latter modified electrode (Fayemi and Adekunle, 2015), probably due to the enhancement of the electron transfer process induced by this type of nanomaterial (Eissa et al., 2019; De Rycke et al., 2020).

The platforms were also assessed via EIS using the same redox probe and the parameters of the equivalent circuit were fitted using NOVA1.10 software. The parameters of the equivalent circuits for the three configurations were summed up in Table 2. As it can be seen from Figure 2B, the circuit on the graphite-based electrode was defined as [R_s_(Q[R_ct_W])] where R_s_ – the resistance of the electrolyte solution, R_ct_ – the charge transfer resistance at the electrode/solution interface, W – the Warburg impedance, Q – constant phase element (CPE), as it was already confirmed by previous studies (Cernat et al., 2018; Cernat et al., 2019) The presence of a Q, meaning the CPE element, is justified by the high porosity of the working electrodes. The equivalent circuit on the GPH electrodes was fitted as [R_s_(C_1_[R_ct_W])(RC_2_)]. In this case CPE was replaced by a classical capacitance – C_1_ suggesting a decrease in the porosity of the working surface and a higher coverage with graphene. A supplementary series of a RC_2_ parallel combination was also inserted, which is characteristic of a system described by a time constant. This fact suggests the presence of the interface between two materials with different properties: the printed graphite layer and the graphene layer deposited on the electrode surface that slows down the access of the electrolyte species at the surface of the electrode for the electron transfer process.

**TABLE 2:** Parameters of the Randles equivalent circuit fitted using NOVA 1.10 software. The numbers in bold represent the charge transfer resistance at the electrode interface in the case of the tested platforms.

Platform	R_s_ (Ω)	R_ct_ (kΩ)	Q/CPE		W (mMho)	C_1_ (µF)	C_2_ (µF)	R (Ω)	χ^2^
			Y0 (µMho)	n					
Graphite-SPE	111	**1.31**	2.72	0.959	1.01	—	—	—	0.0311
GPH	93.8	**0.21**	—		1.17	357	66.2	12.3	0.0132
MWCNTs	69.7	**0.19**	532	0.437	2.38	—	68	3.45	0.0006

In the case of the MWCNT-based platform, the equivalent circuit [R_s_(Q[R_ct_W])(RC_2_)] involves also a supplementary series of a RC_2_ parallel combination along with the circuit determined on the graphite-based electrode. It could be explained by the fact that the morphology of the MWCNTs allows a better access of the electrolyte species to the working surface of the electrode in comparison with the GPH platforms. The replacement of C with the Q element suggests a higher porosity generated by the tubular structure of the MWCNTs.

It can be also observed that the χ^2^ values are low for all three proposed circuits, so it can be affirmed that these models are suitable for modeling processes on the electrode surface.

When assessing the variation of R_ct_ it can be observed that lower values were determined for the GPH and MWCNT configurations suggesting a better electronic transfer rate in the presence of the carbon-based nanomaterials.

### (Pre)Treatment Step

The chosen platforms underwent a (pre)treatment to increase their analytical performances and to be successfully applied for the simultaneous detection in the presence of the chosen adulterants. The electrochemical procedure in 0.5 M H_2_SO_4_ has the purpose to activate the working surface and to remove any impurities that could have a signal in the tested potential range. The Tween 20 conditioning reduces the hydrophobic effect of the electrode surface and allows the access of electrochemically active species from solutions to the pores.

**FIGURE 3:**
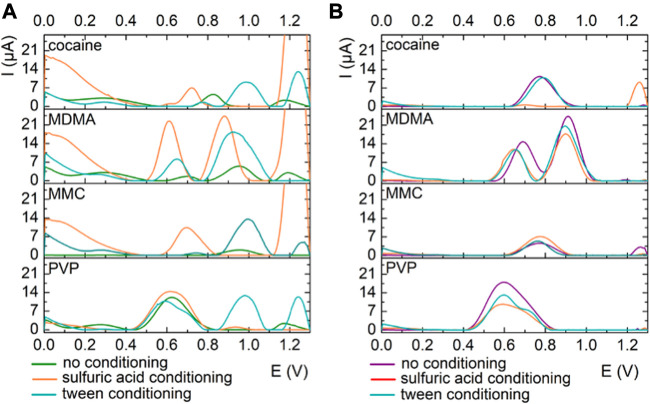
Electrochemical behavior of the illicit drugs performed in a 0.5 mM solution of each illicit drug in PBS pH 12 on **(A)** GPH electrode and **(B)** MWCNTs electrode with no pretreatment (black on SPE-GPH/purple on SPE-MWCNTs), after pretreatment in 0.5 mM H_2_SO_4_ conditiong of the electrodes (orange) and after adding 1% Tween 20 to the electrolyte (turquoise).

Thus, the GPH and MWCNT-based platforms were tested in the presence of the four illicit drugs molecules as presented in Figure 3. For the MWCNT-based platform no significant current increase was observed after any of the two strategies (electrochemical treatment in H_2_SO_4_ electrolyte/Tween 20 conditioning). In the case of the GPH platform the conditioning in H_2_SO_4_ led to higher analytical signals along with a cathodic shift suggesting an improvement in the electronic transfer after the pretreatment for cocaine, MDMA, MMC, and only a slight increase for PVP. The Tween 20 conditioning determined generally an increase of the signal inferior to the one described by the first strategy. In the case of MDMA, MMC an increase of the current was observed in the same conditions as for cocaine, while for PVP the intensity of the peak found at approximately 0.65 V was not influenced. For PVP it was observed a supplementary peak like a shoulder on the main signal and this could mean an enriched fingerprint of this analyte. The purpose of this work is to assess the optimal configuration for all the studied compounds, thus this observation will be exploited on further studies focused only on PVP.

Based on the optimization experimental studies, the platforms based on GPH and MWCNTs were chosen for the analysis of cocaine, MDMA, MCMC and PVP at pH 12 without any additional pretreatment.

Evaluating the overall data obtained on both platforms it was decided to avoid any pretreatment protocols for the following experiments in which adulterants and binary mixtures of illicit drugs and adulterants were analyzed. The reason for that is that any (pre)treatment performed, despite its advantage, involves a supplementary step that is both time consuming and complicated when the sensors would be applied on-site for real street samples and manipulated by non-specialized end-users.

### Evaluation of the Electrochemical Behavior of Some Adulterants on the Optimized Platforms

The same protocol and optimized conditions as described above for the testing of the four illicit drugs molecules was performed on the most common adulterants found in street samples based on the literature studies (Kudlacek et al., 2017; Fiorentin et al., 2019), namely lactose, benzocaine, caffeine and acetaminophen.

Lactose, that is used as a diluting compound, without any pharmacological activity had no oxidation signal in the optimized described conditions, this being an advantage for the previewed purpose of these platforms, namely detection of real samples.

Benzocaine, caffeine and acetaminophen are used to add a boosting effect to the one of the illicit drug and also as diluting agents (Cole et al., 2011; Fiorentin et al., 2019) to reduce the percentage of the psychoactive molecules without reducing the effect. The experimental data showed that benzocaine and acetaminophen had oxidation potentials at ∼0.52 V and ∼0.07 V respectively, these being much lower than the ones obtained for the illicit drugs and in consequence a lower chance to overlap their analytical signal. The oxidation potential of caffeine was registered at 0.99 V on GPH-based platform, higher than the ones registered for the drugs on the same configuration (∼ 0.77 V for cocaine, ∼ 0.64 V for MDMA, ∼ 0.72 V for MMC and ∼ 0.54 V for PVP), while no signal was determined on the MWCNTs for this analyte.

The electrochemical parameters corresponding to the oxidation of the chosen adulterants molecules were summarized in Table 3.

**TABLE 3:** The influence of the type of platform on the electrochemical oxidation of adulterants at pH 12.

Platform	Lactose^a^		Benzocaine		Caffeine		Acetaminophen	
	E (V)	I (µA)	E (V)	I (µA)	E (V)	I (µA)	E (V)	I (µA)
GPH	—	—	0.51	11.74 ± 0.68	0.99	27.78 ± 0.62	0.06	29.77 ± 1.25
MWCNTs	—	—	0.52	15.22 ± 1.12	—	—	0.07	43.11 ± 2.99

^a^ No electrochemical signal.

### Evaluation of Binary Mixtures

**FIGURE 4:**
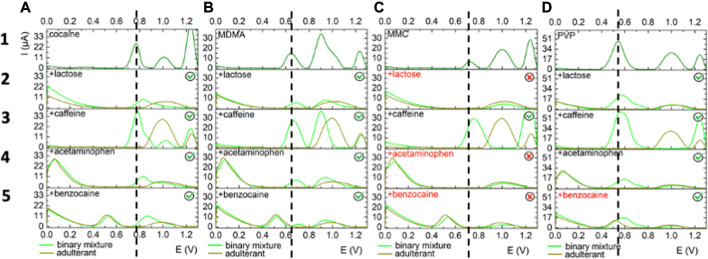
Electrochemical behavior on GPH electrodes of (i) each illicit drug **(A)** cocaine, **(B)** MDMA, **(C)** MMC, **(D)** PVP) in 0.5 mM standard solution - upper first layer (dark green); (ii) each adulterant/cutting agent (lactose, caffeine, acetaminophen and benzocaine) in 0.5 mM standard solution - 2nd to 5th layer (olive green) and (iii) equimolar binary mixtures of illicit drugs and adulterants/cutting - 2nd to 5th layer (light green). The dashed line indicates the oxidation peak potential corresponding to the illicit drug without the adulterants.

**FIGURE 5:**
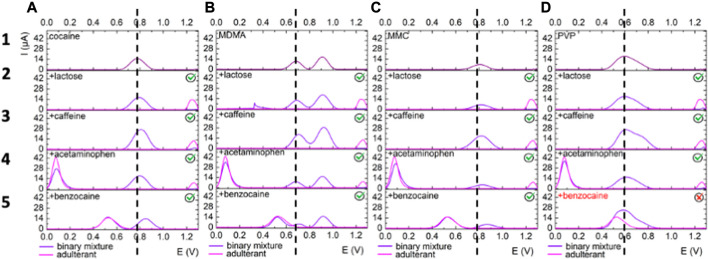
Electrochemical behavior on MWCNTs electrodes of **(I)** each drug of abuse **(A)** cocaine, **(B)** MDMA, **(C)** MMC, **(D)** PVP)in 0.5 mM standard solution - upper first layer (purple); (ii) each adulterant/cutting agent (lactose, caffeine, acetaminophen and benzocaine) in 0.5 mM standard solution - 2nd to 5th layer (pink) and (iii) equimolar binary mixtures of illicit drugs and adulterants/cutting - 2nd to 5th layer (magenta). The dashed line indicates the oxidation peak potential corresponding to the illicit drug without the adulterants.

To have insight regarding the influence of the studied adulterants on the electrochemical detection of cocaine, MDMA, MMC, and PVP, binary mixtures of each target illicit drug and adulterant in equimolar ratio (0.5 mM : 0.5 mM) were analyzed on each platform at pH 12, as discussed in the experimental part. The data (baseline corrected using the moving average filter from Nova 1.10 software (window size 1)) for GPH/MWCNT-based platforms are presented in Figures 4, 5 and the analytical parameters concerning the modifications in current intensity and potential shift are summarized in Table 4 to offer a broader perspective on their mutual influence.

**TABLE 4:** Variation of the analytical performances of cocaine, MDMA, MMC and PVP depending on the platform and adulterant molecule. The values in bold represent the current intensity and potential values for the tested pure drugs on different platforms.

Platform	Mixture components	Cocaine		MDMA		MMC		PVP	
		E (V)	I (µA)	E (V)	I (µA)	E (V)	I (µA)	E (V)	I (µA)
**GPH**	Pure drug	**0.77**	**26.07** ± 0.75	**0.63**	**14.81** ± 1.26	**0.72**	**6.57** ± 0.36	**0.54**	**47.33** ± 0.94
	+Lactose	0.83 →	8.82 ± 0.03↓	0.69 →	5.11 ± 0.14↓	—	—	0.57 →	23.6 ± 0.05↓
	+Caffeine	0.78	38.22 ± 0.58↑	0.66 →	26.8±0.39↑	0.76 →	29.62 ± 1.61↑	0.56 →	62.8 ± 0.59↑
	+Acetaminophen	0.83 →	11.01 ± 0.01↓	0.69 →	7.10 ± 0.03↓	—	—	0.59 →	15.23 ± 0.10↓
	+Benzocaine	0.86 →	10.92 ± 0.04↓	0.72 →	1.99 ± 0.04↓	—	—	0.58	16.71 ± 1.24↓
**MWCNTs**	Pure drug	**0.77**	**15.04** ± 0.33	**0.68** **0.91**	**10.82** ± 0.49 **16.69** ± 0.91	**0.81**	**6.76** ± 0.46	**0.59**	**17.98** ± 1.40
	+Lactose	0.79	16.33 ± 0.01↑	0.68 0.91	10.2 ± 1.14↓ 17.8 ± 1.35↑	0.82	6.10 ± 0.26↓	0.59	17.261.73↓
	+Caffeine	0.81 →	27.01 ± 1.64↑	0.70 → 0.92 →	15.2 ± 1.14↑ 25.4 ± 1.05↑	0.82	17.75 ± 1.66↑	0.60	26.35 ± 1.53↑
	+Acetaminophen	0.79 →	17.68 ± 1.35↑	0.68 0.91	7.82 ± 0.1↓ 15.2 ± 1.19↓	0.82	4.90 ± 0.19↓	0.61	15.39 ± 0.01↓
	+Benzocaine	0.84 →	13.73 ± 0.37↓	0.71 → 0.91	3.17 ± 0.14↓ 14.8 ± 0.7↓	0.87 →	5.04 ± 0.16↓	0.58	24.59 ± 1.23↑

#### Cocaine

On the GPH modified electrode, lactose, acetaminophen and benzocaine caused a decrease in the current intensity of the peak registered at 0.77 V for cocaine, while in the presence of caffeine, the intensity of the peak was almost 50% increased (Figure 4A). On the second platform, lactose and acetaminophen had no influence on the oxidation peak of cocaine (determined at 0.775 V when analyzed alone) from both the potential and current intensity perspectives and benzocaine determined an anodic shift of approximately 80 mV (de Jong et al., 2020). Caffeine determined a two times fold increase in the current intensity of the oxidation peak (Figure 5A).

#### MDMA

The intensity of the peak registered at 0.639 V on the GPH-based electrode registered an anodic shift of 30–60 mV in the presence of lactose, acetaminophen and caffeine, and 80 mV when analyzed in the presence of benzocaine. Regarding the current intensity, it was severely diminished by all adulterants with the exception of caffeine where it was determined a two times fold increase, following the same behavior as in the case of cocaine (Figure 4B).

On MWCNT electrode lactose, acetaminophen, and benzocaine had no major influence on the potential of the two peaks and a slight decrease in the current intensity was observed. As for caffeine, the same trend was followed and the current intensity had a 50% increase, as it can be seen from Figure 5B.

#### MMC

A 40 mV shift in the peak position of MMC in the presence of caffeine was observed on GPH electrodes. As expected the current intensity corresponding to the electrochemical oxidation of the molecule was 4 times fold enhanced in the presence of caffeine (Figure 4C). In this case no signal was observed in the presence of lactose, acetaminophen, and benzocaine that completely blocked the electrochemical signal of MMC (Figure 4C). On MWCNTs, no potential was observed in the presence of lactose, caffeine and acetaminophen, while in the presence of benzocaine the potential shift was of 60 mV (Figure 5C). Regarding the current intensity for MMC oxidation, the signal was decreased in all the cases except caffeine where it was generated an increase of about 3 times fold.

#### PVP

Lactose and acetaminophen determined a decrease of the current intensity, while caffeine, as usual, caused an increase on both GPH and MWCNTs platforms (Figure 4D and Figure 5D). As for benzocaine, the signal overlapped with the one of PVP and it was not possible to be separated in the studied conditions.

Overall, the four studied molecules were detected in the presence of the analyzed adulterants with some exceptions where the signal of MMC on GPH was blocked by lactose and acetaminophen through some form of suppression and by benzocaine through overlap of signals, and also the signal of PVP on both platforms by benzocaine through overlap of signals as well. Of course, there were shifts in the peak potentials and variations in the current intensity when comparing with the single illicit drugs tests, but minimum overlapping was noted, an important advantage in the detection of multiple illicit drugs.

In this case, the sensors could be used for a rapid qualitative screening of potential real samples. The quantitative assays of the drugs in the presence of the studied adulterants are difficult to achieve due to their mutual influence when analyzed at the concentrations of 0.5 mM. Caffeine is a psychostimulant compound and it is frequently used as an adulterant in association with cocaine and it has the capacity to potentiate the stimulant effects of cocaine and cocaine-induced drug seeking behavior (Muñiz et al., 2017). For instance, caffeine is oxidized to a 4,5-diol analogue compound and only a slight shift was reported when the ratio illicit drug : adulterant was 2, and not 1, like in this study. Moreover, previous studies reported that the concentration of caffeine was inferior to the one of cocaine (and not an equimolar ratio like in this work) and this could explain the increase in current intensity of the cocaine peak. The same trend was observed on all the studied molecules due to their interaction, that are not clear at this point (De Jong et al., 2016).

The shift of the oxidation potential corresponding to the electrochemical oxidation of the illicit drugs in binary mixtures could be easily spotted when following the dash line characteristic for the compound analyzed without the adulterants. Also the variation in the current intensity is followed based on the direction of the arrows included in Table 4.

### Assessment of Real Samples

The four target molecules were also assessed in real samples, such as tap water and waste water (van Nuijs et al., 2018) on both platforms: GPH and MWCNTs. As described in the experimental part, the samples were diluted with PBS in 1:1 ratio and pH was kept at 12 to meet the optimized parameters. The results were compared with the data obtained on the analysis of single compounds and recovery rates were calculated for the corresponding current intensity and summarized in Table 5. As it can be seen the recovery rate for the experiments performed in tap water diluted 1: 1 with phosphate buffer solution of pH 12, generally ranged from 98%-117%, with RSD from 1-8%, that can be considered acceptable for this type of assessment. The MMC generated a low recovery rate in GPH-based platforms, but on MWCNTs the results are promising and this approach can be further developed. In the case of waste water samples diluted 1:1 with phosphate buffer solution of pH 12, higher values (112%-146%, RSD 0.7-5%) were obtained due to the existence of other electrochemical species that are prone to be oxidized and the results could be false positive. An additional dilution of the buffer samples is not justified considering that in waste water samples the amount of drug that can be found is very small. It is important to notice, that the matrix effect of the waste water is important due to the complexity of the sample that could contain other electroactive molecules.

**TABLE 5:** Recoveries obtained after the assessment of the four illicit drugs in tap and waste water diluted in a 1:1 rato with PBS pH 12. The data in bold represent the recovery rate for all the tested substances on different platforms.

Analyte	Platform	Standard (PBS pH 12) I (µA)	Tap water I (µA)	Recovery (%)	RSD (%)	WastewaterI (µA)	Recovery (%)	RSD (%)
Cocaine	GPH	26.07	25.57	**98.08**	1.99	38.30	**146.92**	3.38
	MWCNTs	15.04	16.09	**106.98**	8.71	21.20	**140.94**	0.98
MDMA	GPH	14.81	17.41	**117.58**	1.50	18.70	**126.29**	0.83
	MWCNTs	10.82	10.95	**101.17**	2.72	13.52	**124.98**	2.05
		16.69	18.73	**112.24**	1.03	22.57	**135.21**	0.77
MMC	GPH	6.57	3.56	**54.13**	4.83	7.70	**117.20**	7.32
	MWCNTs	6.76	6.78	**100.30**	3.19	7.61	**112.57**	1.58
PVP	GPH	47.33	54.13	**114.36**	2.47	48.90	**103.32**	3.76
	MWCNTs	17.98	17.22	**95.77**	1.52	20.55	**114.31**	4.99

## Conclusion and Perspectives

This extensive study determined the optimized platforms and experimental conditions to assess some molecules of illicit drugs, namely cocaine, MDMA, MMC and PVP, alone and in the presence of commonly adulterants found in real samples (caffeine, acetaminophen, lactose and benzocaine). Thus, GPH/MWCNT-based platforms proved to have the best results at pH 12 in comparison with the unmodified graphite SPE or modified with electrochemically generated Au/PtNPs. Despite the increase in current intensity for the illicit drugs observed after the pretreatments in 0.5 M H_2_SO_4_ and Tween 20 these strategies were not applied due to the previewed outcome related to the development of portable devices. Moreover, a complicated protocol was avoided that could contribute to an increase of the compliance of nonspecialized users as the platforms are intended to be further integrated in portable devices for on-site use.

When assessed in the presence of adulterants it was clearly observed a mutual influence that makes the quantitative detection difficult to achieve, without a chemometric interpretation. Hence, the sensors could be applied for the on-field rapid screening of samples that is suitable for first responders to allow a better management of these situations.

Further studies are necessary to assess the presence of illicit drugs in tertiary/quaternary mixtures *via* multivariate analysis and to evaluate real street samples for the development of an integrated sensing device.

## Data Availability

The raw data supporting the conclusions of this article will be made available by the authors, without undue reservation.
